# Wireless Remote Monitoring of Toxic Gases in Shipbuilding

**DOI:** 10.3390/s140202981

**Published:** 2014-02-14

**Authors:** Carlos Pérez-Garrido, Francisco J. González-Castaño, David Chaves-Diéguez, Pedro S. Rodríguez-Hernández

**Affiliations:** AtlantTIC, Universidade de Vigo, EI Telecomunicación, Rúa Maxwell S/N, Vigo 36310, Spain; E-Mails: cpgarrido@enigma.det.uvigo.es (C.P.-G.); dchaves@gradiant.org (D.C.-D.); pedro@det.uvigo.es (P.S.R.-H.)

**Keywords:** WSN, IEEE 802.15.4, Zigbee, wireless communications in confined spaces, shipbuilding

## Abstract

Large-scale wireless sensor networks have not achieved market impact, so far. Nevertheless, this technology may be applied successfully to small-scale niche markets. Shipyards are hazardous working environments with many potential risks to worker safety. Toxic gases generated in soldering processes in enclosed spaces (e.g., cargo holds) are one such risk. The dynamic environment of a ship under construction makes it very difficult to plan gas detection fixed infrastructures connected to external monitoring stations via wired links. While portable devices with gas level indicators exist, they require workers to monitor measurements, often in situations where they are focused on other tasks for relatively long periods. In this work, we present a wireless multihop remote gas monitoring system for shipyard environments that has been tested in a real ship under construction. Using this system, we validate IEEE 802.15.4/Zigbee wireless networks as a suitable technology to connect gas detectors to control stations outside the ships. These networks have the added benefit that they reconfigure themselves dynamically in case of network failure or redeployment, for example when a relay is moved to a new location. Performance measurements include round trip time (which determines the alert response time for safety teams) and link quality indicator and packet error rate (which determine communication robustness).

## 1. Introduction

Large-scale wireless sensor networks have not achieved market impact, so far. Indeed, it has been stated that wireless multihop technology is impractical [1]. Nevertheless, this technology may be applied successfully to small-scale niche markets, such as personal safety.

Although there is a scarcity of information about work accidents in confined spaces, such as shipbuilding facilities, several studies report that about 6,000 serious injuries and between 40 and 65 deaths occur each year in these environments [2]. One of the main causes of accidents and fatalities in enclosed industrial workplaces is the buildup of dangerous gases. Many accidents are due to toxic/asphyxiating atmospheres [2]. Reports by OSHA (United States Occupational Safety and Health Administration) and MSHA (United States Mine Safety and Health Administration) agree that the main cause of injuries and deaths in those scenarios is inadequate control of dangerous activities.

Three potential hazards are generally considered in workspace atmosphere monitoring: (a) oxygen depletion; (b) toxic gases; and (c) combustible gases. In shipbuilding, toxic gases from soldering and thermal cutting processes are the main source of accidents and are associated with many adverse health effects [3].

In a finished ship, gas detectors are placed in carefully planned locations. These detectors are attached to the wired network of the vessel. In shipbuilding, however, gas monitoring is much more difficult, due to the dynamic working environment, the lack of wired networks, the practical impossibility of laying these networks and the presence of metal structures throughout the ship that hinder radio communications. Portable gas detectors provide an alternative means for measuring gas concentrations manually at work spots. Companies, such as Dräger [4], MSA (Mine Safety Appliances) [5] and Casella [6] produce small portable detection devices whose readings need to be checked frequently, as dangerous atmospheres develop quickly. Such readings typically need to be monitored by workers, who are frequently focused on other tasks for relatively long periods. In this regard, it has been demonstrated that delegating safety measures to workers in highly complex environments is a very dangerous practice. Previous studies [7] have shown that:

- About 65% of all fatalities in confined spaces involve workers who were unaware that the space they were entering was hazardous, and over 50% of these workers were members of rescue teams.
- Over one third of the fatalities occurred in spaces that had been declared safe after testing with a gas detector that had been moved elsewhere.

Current safety protocols define the maximum times of exposure and concentrations for different gases [8,9]. Exposure to Class A hazards (IDLH or immediately dangerous to life or health) is highly restricted. However, safety protocols determine that a person may be confined in a space with IDLH toxic gases for several minutes. For lower hazard levels, a worker may spend up to 8 h in a space with potentially dangerous gases.

Taking these references into account, we defined a desired response time of less than a minute. This is negligible according to current regulations.

Given its flexibility, our system could be used in the construction of a wide variety of ships, ranging from research vessels (RV, with a length of 100 m, a beam of 20 m and a draft of 10 m, as in the field tests in Section 4) up to ultra-large crude carriers (ULCC, with a length of 400 m, a beam of 60 m and a draft of 40 m).

Project GasVivo by Universidad de Vigo and Idega Preventión, Spain (this research has been funded by grants PGIDIT10DPI080E, Xunta de Galicia, Spain, and TEC2010-21405-C02-01, MINECO, Spain) was designed to develop a wireless multihop toxic gas remote monitoring system, with the following basic requirements: (*i*) the multihop network must reconfigure itself in case a monitoring or relay node is taken to another place; (*ii*) a control station outside the ship must be permanently aware of the measurements; and (*iii*) in case of danger, the response time of the system must be negligible for the safety protocols in place.

The main objectives of our research were to evaluate the feasibility of wireless sensor networks to transmit measurements to monitoring stations outside a large ship under construction, and, in particular, to validate our solution in a real working environment.

The data sources in our system were Dräger X-am 5000 portable gas detectors [10]. These units can store around 1,000 h of measurements of five gases, with a sampling interval of one reading per minute. Nevertheless, they can also deliver readings in real-time through an infrared interface. We extended the detectors with IrDA-RS232 adaptation boards to connect them with Zigbee end devices (ZED).

Several tests were conducted to compare the performance of the system with the results obtained in near-free space conditions. The tests measured the RTT (round trip time), PER (packet error rate) and LQI (link quality indicator) of wireless connections, taking into account the number of hops the packets needed to reach their destination. The final quality metric were the response time of the system, from the point of view of a control station outside the ship, and its robustness.

The rest of this paper is organized as follows: Section 2 reviews related work in communications in confined spaces and ship environments. Section 3 describes our system and the topology and methods used for the measurements. Section 4 discusses the results, and finally, Section 5 concludes the paper.

## 2. Previous Work

There is plenty of work on wireless sensor networks for environmental and gas monitoring [11-14], as well as on wireless propagation in diverse indoor [15-17] and outdoor [18] conditions. The case of hazardous confined spaces, such as underground mining galleries and railroad tunnels, has also been studied [19,20].

Some authors have considered static wireless networks as an alternative to wired networks in finished vessels. For example, Paik *et al.* [21] and Pilsak *et al.* [22] proposed wireless sensor networks for communication with the main engine rooms of ships in active service, focusing on available technologies, and Zaharia *et al.* [23] proposed Zigbee for in-deck communications. They also described some models to estimate path loss in different ship locations.

Kdouh *et al.* [24] performed tests on the communication range of nodes within a vessel. They verified that radio links could not be established between different decks or even different ship compartments without a clear connecting path. Park *et al.* [25] arrived at a similar conclusion. They evaluated Zigbee wireless transmissions through several steel plates commonly found in a ship (1.6-mm steel plates, 1-mm steel plates and 0.4-mm galvanized steel plates). According to their results, communication was only possible for the thinnest plates, and the PER was very high in that case. Hence, direct line-of-sight paths are essential for connecting different decks and compartments in a vessel.

Previous work has considered Zigbee and Bluetooth technologies for in-ship crew location and telemonitoring [26,27]. However, neither these nor the above works considered ships under construction. This environment is intrinsically dynamic and does not allow for fixed networks, due to the presence of heavy machinery and metal panels that appear and disappear.

In a finished ship, wireless communications may be an alternative to wired ones to reduce costs, but in ship construction they are the only alternative at hand. It is simply impossible to lay wires between communication nodes, because they can be cut, and the nodes themselves are taken from one place to another as the ship is built.

## 3. Systems and Methodology

### 3.1. Systems

The gas monitoring system built for this study had three main subsystems: a data acquisition subsystem, a data processing subsystem and a data transport subsystem.

The data acquisition subsystem, as previously mentioned, was built around a Dräger X-am 5000 portable gas detector, which we extended with a RS232-to-infrared conversion adapter to connect it to a ZED.

The data processing subsystem was located in the central monitoring station of the shipyard. Its main function was to gather gas level readings from the Zigbee network coordinator (to which it was directly connected via RS232), process these readings and generate alarms if a potentially hazardous situation was detected.

Finally, the data transport subsystem provided a wireless bidirectional channel between the data acquisition and data processing subsystems. We selected IEEE 802.15.4/Zigbee as the supporting technology for this subsystem for the following reasons:

- Zigbee can operate in several different Industrial, Scientific and Medical (ISM) bands (2.4 GHz worldwide; 868 MHz in Europe; 915 MHz in the US).
- Theoretically, the Zigbee protocol allows up to 16,000 networks to coexist on the same channel, with up to 65,000 devices in each of them.
- Zigbee is a multihop communication protocol with dynamic mesh routing. It is intrinsically survivable, in the sense that communication paths automatically recover from node failures. Routes change dynamically in such a case.
- Zigbee low power communications allow sensing nodes to remain active for long periods of time without maintenance, with node autonomy easily lasting a whole working day.

Since the data acquisition and data processing subsystems could be easily validated in laboratory conditions, this paper focuses mainly on the data transport subsystem and the challenges to create a wireless communication network in such a complex environment as a ship under construction.

**Figure 1.**
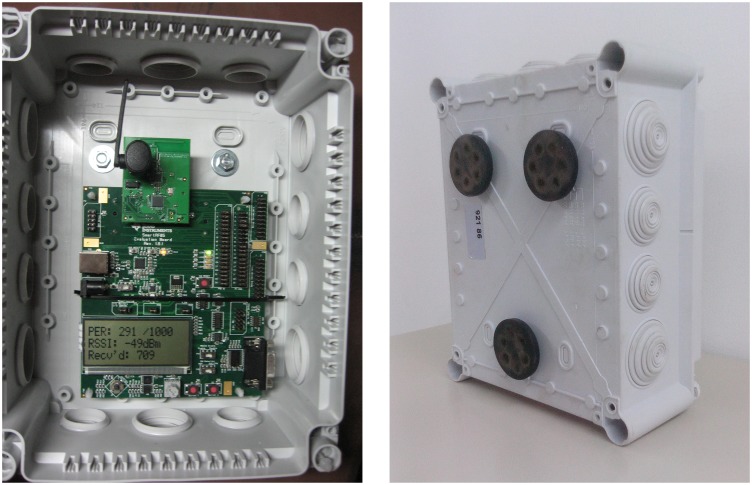
The CC2530-CC2591EM device with TISmartRF05EB board (**a**) and Legrand IP55 PVC boxes with magnetic fixing (**b**).

The system in this research comprised six gas monitoring nodes. Each included a Texas Instruments CC2530-CC2591EM device [28] mounted on a TISmartRF05EB board (Figure 1a), with protocol stack ZigBee Pro Z-Stack version 2.5.1a [29]. One of the devices was loaded with Zigbee Coordinator (ZC) firmware, another one with ZED firmware, and the other four with Zigbee Routers (ZR). The nodes were encased in Legrand IP55 polyvinyl chloride (PVC) boxes with magnetic fixing (Figure 1b), which allowed them to be attached to metal walls.

The Dräger X-am 5000 portable gas detector and the Zigbee kit cost € 1,000 and € 800, respectively. Some additional € 300 were spent on IrDA-RS232 adapters and enclosures. Therefore, the total cost of each measuring point in a ship under construction would be approximately 18% more than the original cost of a single portable gas detector.

This can be considered reasonable given the improvement in functionality. It should be recalled, however, that the primary objective of this work was not to find a cost-effective solution, but to validate the feasibility of wireless gas monitoring in shipyard environments. The optimization of device costs and sizes can be explored in future work.

### 3.2. Methodology

Two types of tests allowed us to evaluate the performance of the system in the target environment: line-of-sight outdoor tests to estimate performance bounds and in-ship real tests. In order to filter the effect of moving machinery and personnel in particular tests, in each experiment, each monitoring point transmitted 1,000 data frames. Each experiment was repeated three times. All the plots in the figures of this paper show mean values and 95% confidence intervals. Single-hop and multihop node locations are shown as blue circles and orange squares, respectively. In all single-hop experiments, only two nodes participated at a time (ZC and another node), whereas in multihop experiments, all available nodes participated.

In single-hop tests, a detector ZC was connected to a PC through RS232. Data frames were sent to a ZED at the maximum transmission power (20 dBm). The ZED then returned each frame received back together with a complementary frame containing the LQI and PER of the messages it received. Finally, the ZC reported the RTT, PER and LQI values for all received packets and the PER and LQI values that the ZED returned. In each single-hop test scenario, the distance between the ZC and the ZED was increased gradually, until communication quality dropped significantly.

Multihop tests were performed in a similar way. However, whenever the distance between the ZC and the ZED increased beyond a point at which they could not see each other, an intermediate ZR was added to the setup, and communications were resumed. Since LQI does not apply to multihop communications, only RTT and PER were reported in these cases.

## 4. Results

### 4.1. Line-of-Sight Outdoor Tests

**Figure 2.**
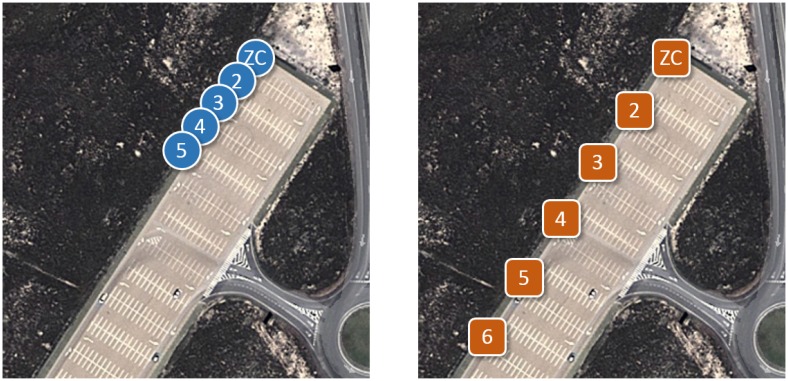
Outdoor scenario, single-hop (**a**) and multihop (**b**) reference tests.

The goal of the line-of-sight outdoor experiments was to evaluate the performance of the system in a favorable environment, as a reference. Outdoor tests took place in a flat open area without obstacles (Figure 2) and with a maximum line-of-sight of 500 m, in order to emulate near-ideal propagation conditions. Transmission power was set to 0 dBm, corresponding to a hop range of about 25 m.

According to the IEEE 802.15.4 specification [30], LQI indicates the strength and quality of the signal received for every data packet. Its values range from zero to 255 (maximum quality). LQI computing depends on the stack implementation. In our case, the experiments showed that LQI varied linearly with RSSI, according to Expression (1), where *a* = 2.62 and *b* = 227.4.

(1) LQI=a⋅RSSI+b

**Figure 3.**
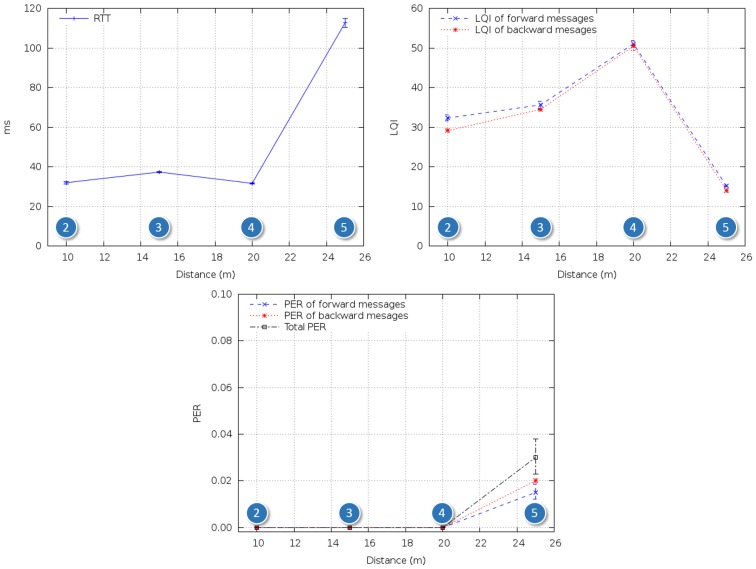
RTT (round trip time), LQI (link quality indicator) and PER (packet error rate), single-hop outdoor reference tests.

Figure 3 shows RTT, LQI and PER in the single-hop tests in the outdoor scenario (Figure 2a). RTT varied between 30 and 40 ms, and no packets were lost, except for the case of the longest hop, in which PER was around 3%. From Equation (1), the corresponding RSSI was approximately -81 dBm, which is relatively close to the nominal sensitivity in the device datasheet.

In order to assess ideal multihop performance, several ZRs were introduced, ensuring that the distance between any two adjacent nodes was within mutual communication range. Figure 2b shows the resulting setup. Adjacent nodes were placed at intervals of 15-20 m, yielding 0% PER and ∼35 ms RTT per hop, which is coherent with Figure 3. RTT and PER were measured for two to six devices, corresponding to one to five hops.

**Figure 4.**
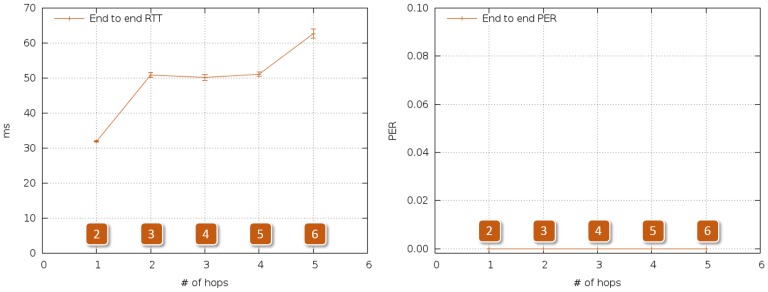
RTT and PER, multihop outdoor reference tests.

Figure 4 summarizes the results of the reference tests. As expected, PER was the same regardless of the number of intermediate nodes, and RTT grew nonlinearly with the number of nodes, reaching 60 ms when the last ZR was added.

### 4.2. In-Ship Tests

**Figure 5.**
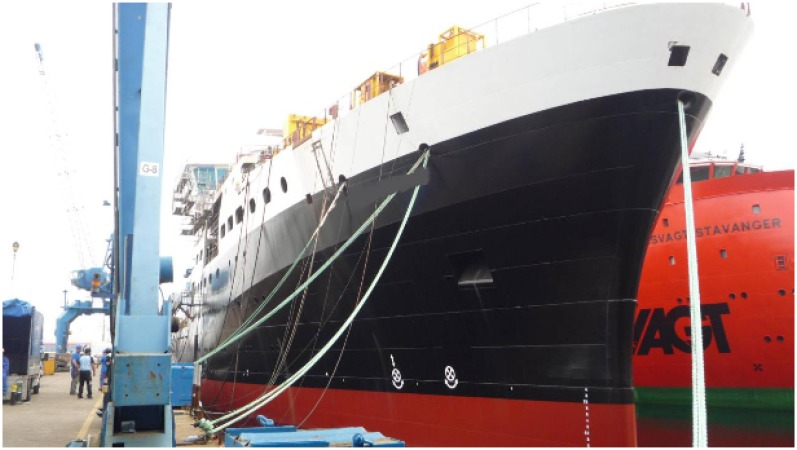
In-ship tests, survey vessel under construction.

In-ship tests took place at *Construcciones Navales Paulino Freire* shipyard, where the survey vessel in Figure 5 was being built at the time of our research. Five different in-ship communication scenarios were considered. In the first four, RTT, LQI and PER of representative internal single-hop links were measured. From that knowledge, for the last scenario, we evaluated PER and RTT for a worst-case multihop path to monitor one of the ship holds, transmitting detector gas readings to a control station outside the vessel. In all the in-ship tests, node transmission power was set to its maximum value (20 dBm).

**Figure 6.**
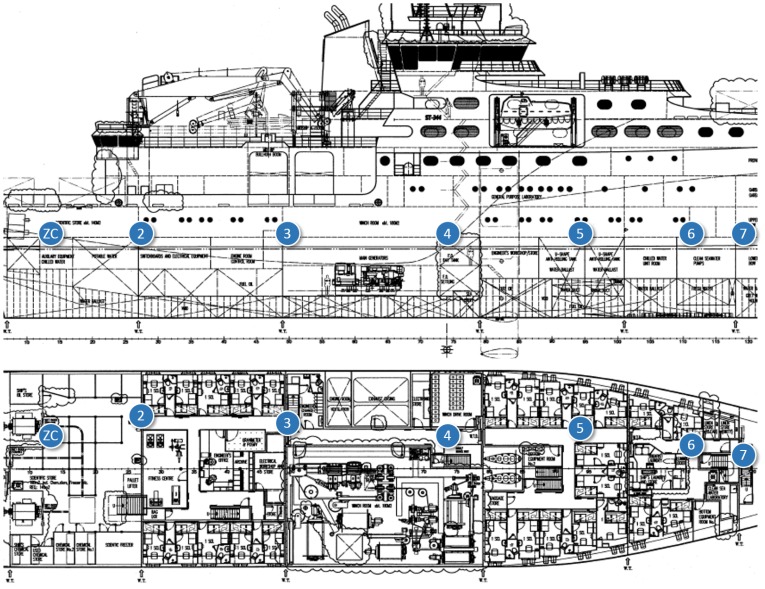
In-ship test Scenario 1: the corridor from stern to prow.

**Figure 7.**
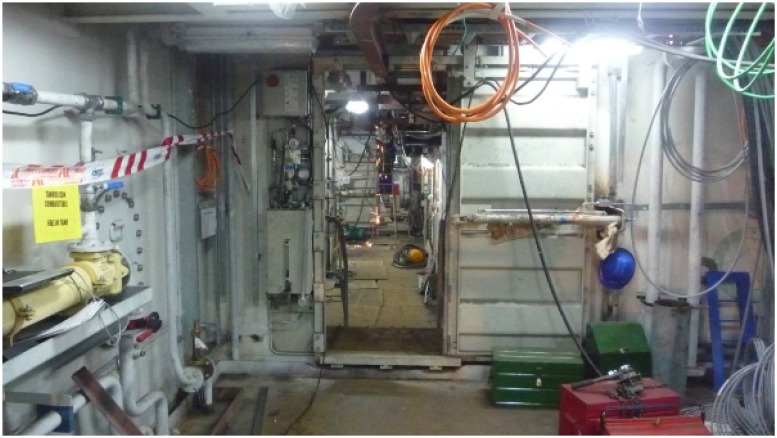
Snapshot of in-ship test Scenario 1.

In the first test scenario (Figures 6 and 7), the nodes were placed along a corridor from stern to prow. The corridor was not completely straight, and there were five doorways blocking the line-of-sight path.

**Figure 8.**
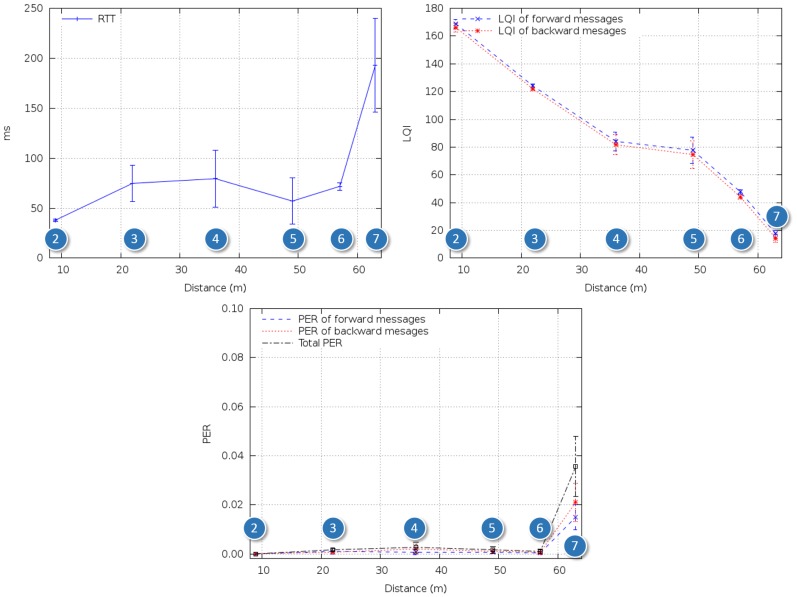
RTT, LQI and PER for in-ship test Scenario 1.

As shown in Figure 8, RTT increased with respect to the reference tests outdoors. This seemed to be mainly due to retransmissions caused by multipath interference and fading produced by workers and equipment passing by. However, LQI stayed within operational range for distances of 60 m, and PER stayed below 3 × 10^-3^ along the path.

**Figure 9.**
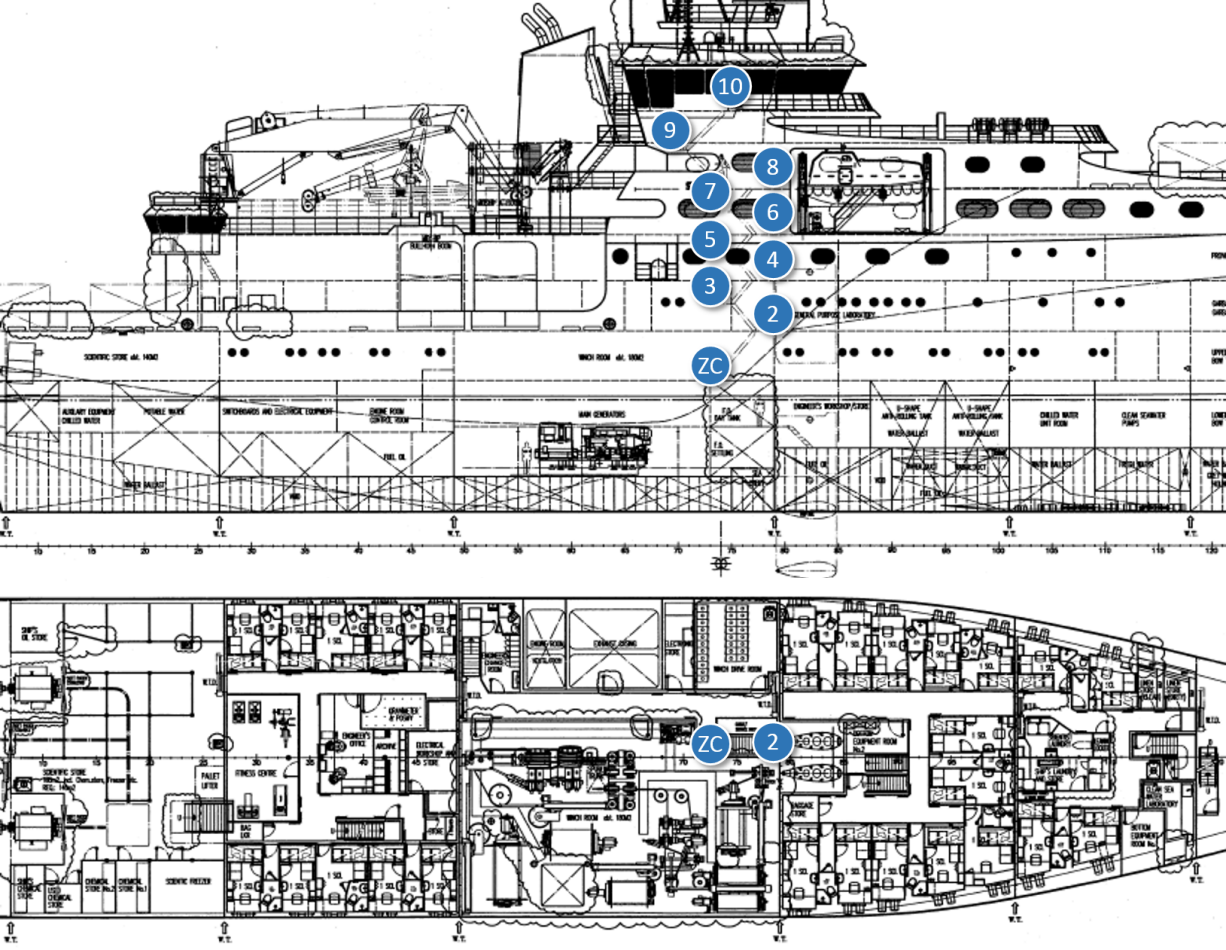
In-ship test scenario #2: Staircase connecting inner decks with bridge.

**Figure 10.**
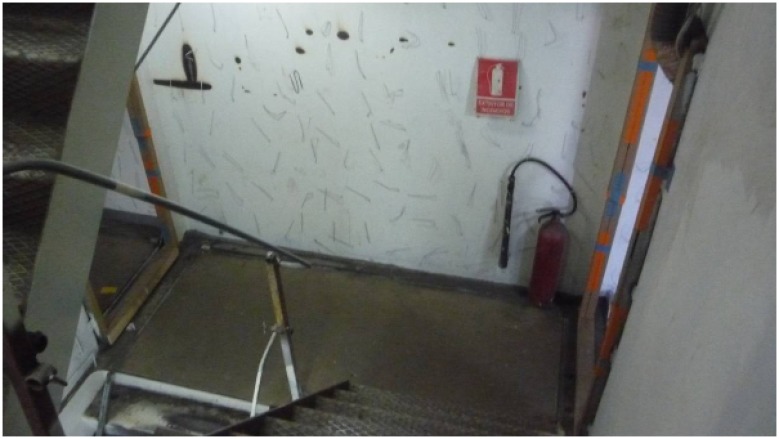
Snapshot of in-ship test Scenario 2.

For the second test scenario (Figures 9 and 10), the nodes were attached to the walls of a staircase that connected the inner decks with the bridge. The ZC was placed at the lowest level and the ZED on successive stairwell landings up to the bridge (between Points 8 and 9 in Figure 9 there is a door and a new staircase begins).

**Figure 11.**
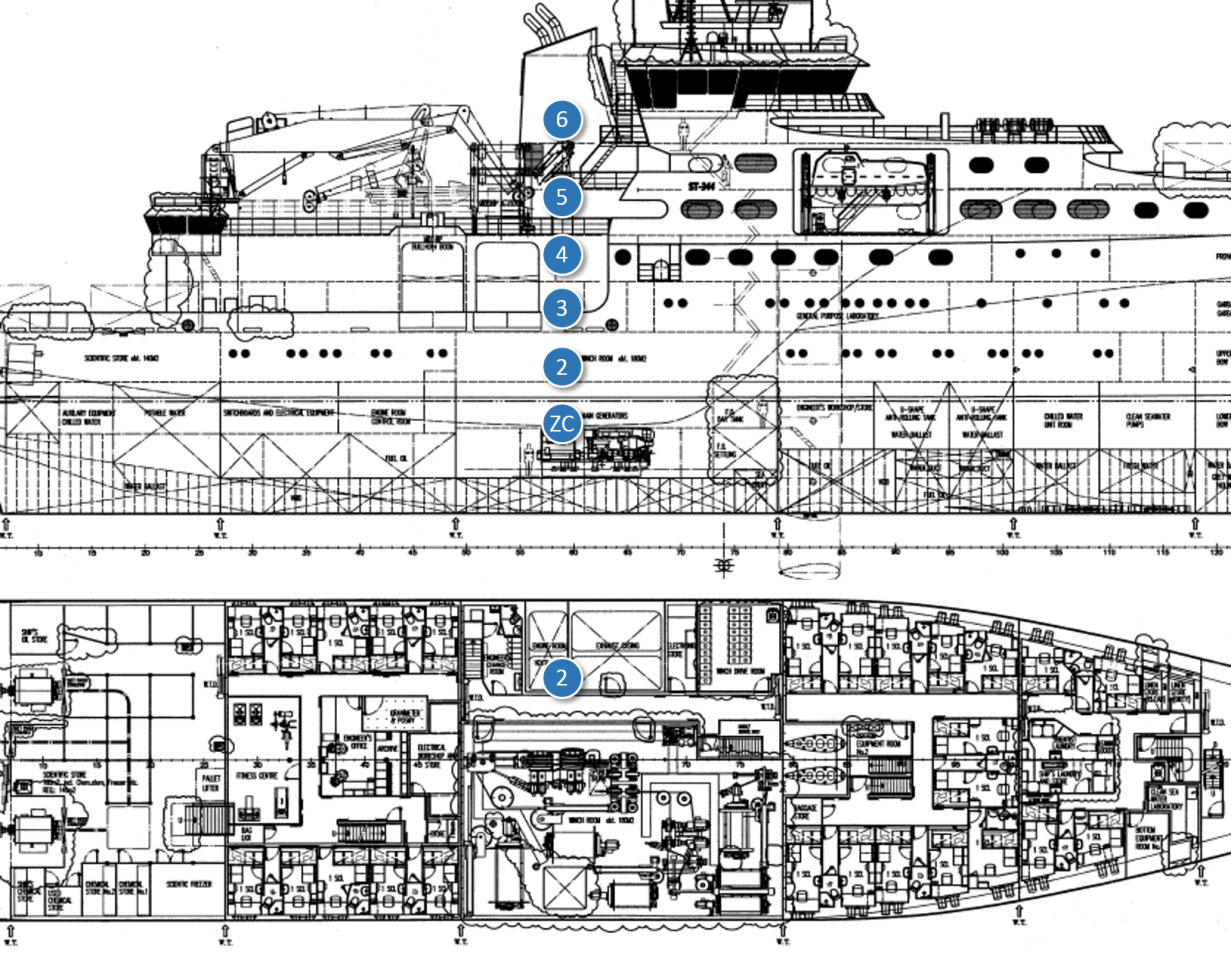
In-ship test Scenario 3: exhaust pipes connecting the engine room with the outside.

**Figure 12.**
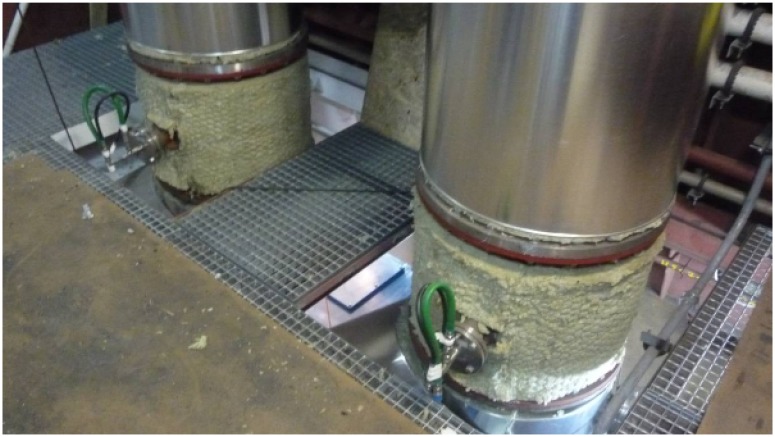
Snapshot of in-ship test Scenario 3.

**Figure 13.**
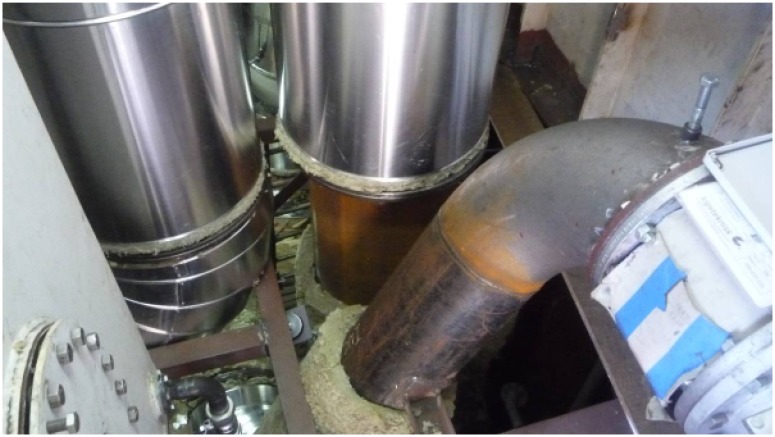
Narrowing near the end of in-ship test Scenario 3.

In the third test scenario (Figures 11 and 12), we tested communications along the space surrounding the exhaust pipe that connected the engine room with the outside. There were metal grids on the floors where the pipe crossed the different levels of the ship, and a substantial narrowing of the space around the pipe near its end (Figure 13).

Characterizing scenarios like 2 and 3 was necessary because, since all floors were metal, radio signals could not propagate from the lower levels unless a clear vertical path was available. In a ship, there are few such spaces, such as staircases and vertical pipes.

**Figure 14.**
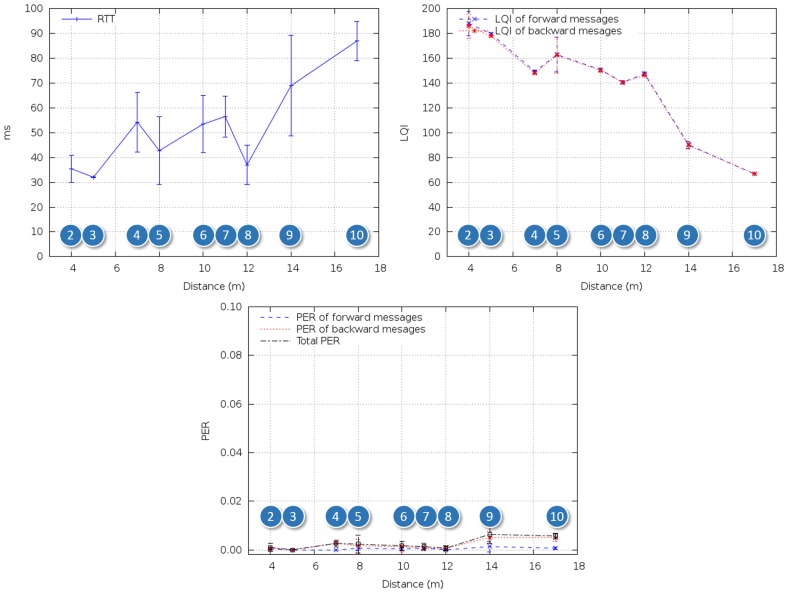
RTT, LQI and PER for in-ship test Scenario 2.

Figure 14 shows the results for Scenario 2. Although it was possible to reach the bridge in a single hop, the effect of the door and staircase change between Points 8 and 9 was clearly perceived as LQI decrease. This decrease conveyed an increase of RTT and PER, but the latter was always below a reasonable value (from the point of view of the end application) of 6 × 10^-3^.

**Figure 15.**
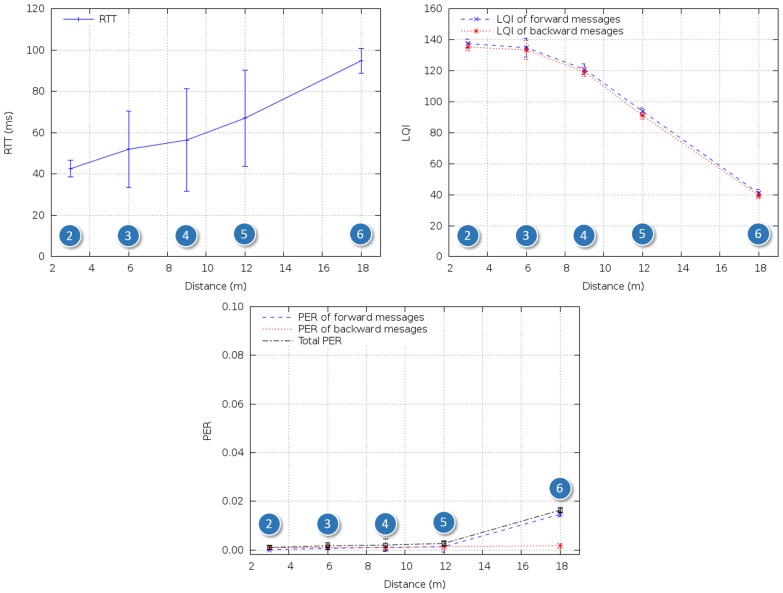
RTT, LQI and PER for in-ship test Scenario 3.

Regarding Scenario 3, as shown in Figure 15, the metal grids separating the different levels of the ship did not significantly affect radio propagation. LQI decreased gradually until the last test point, and RTT and PER varied accordingly. There was a sudden increase in PER at the last test point, possibly due to the narrowing around the pipe.

**Figure 16.**
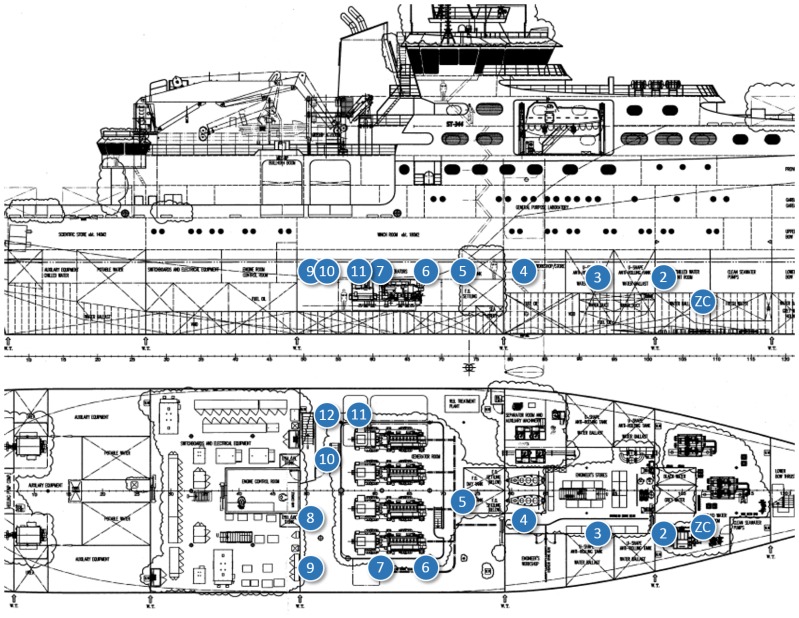
In-ship test Scenario 4: communications between a hold and the engine room of the vessel.

**Figure 17.**
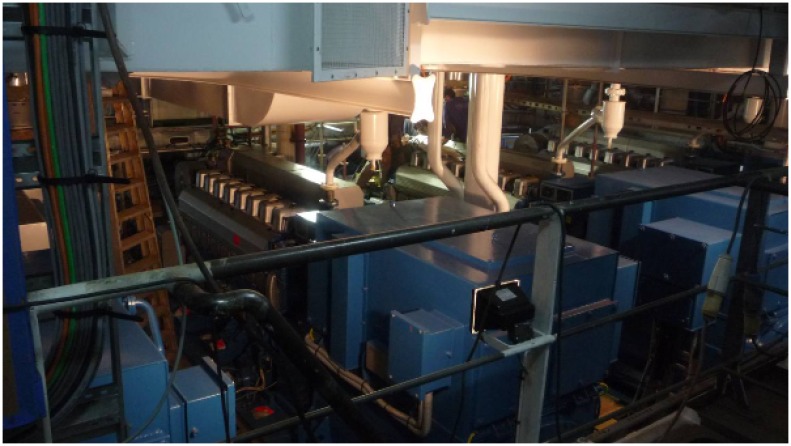
Snapshot of in-ship test Scenario 4.

Figure 16 shows the location of the test points for Scenario 4. The goal of this test was to check if communications were feasible inside the engine room. This room was cramped with machinery, with little free space near the ceiling (Figure 17); furthermore, workers were frequently present there during the construction of the ship. The layout of this room was also very different from that of the rooms on the upper decks and, therefore, justified an *ad hoc* test.

**Figure 18.**
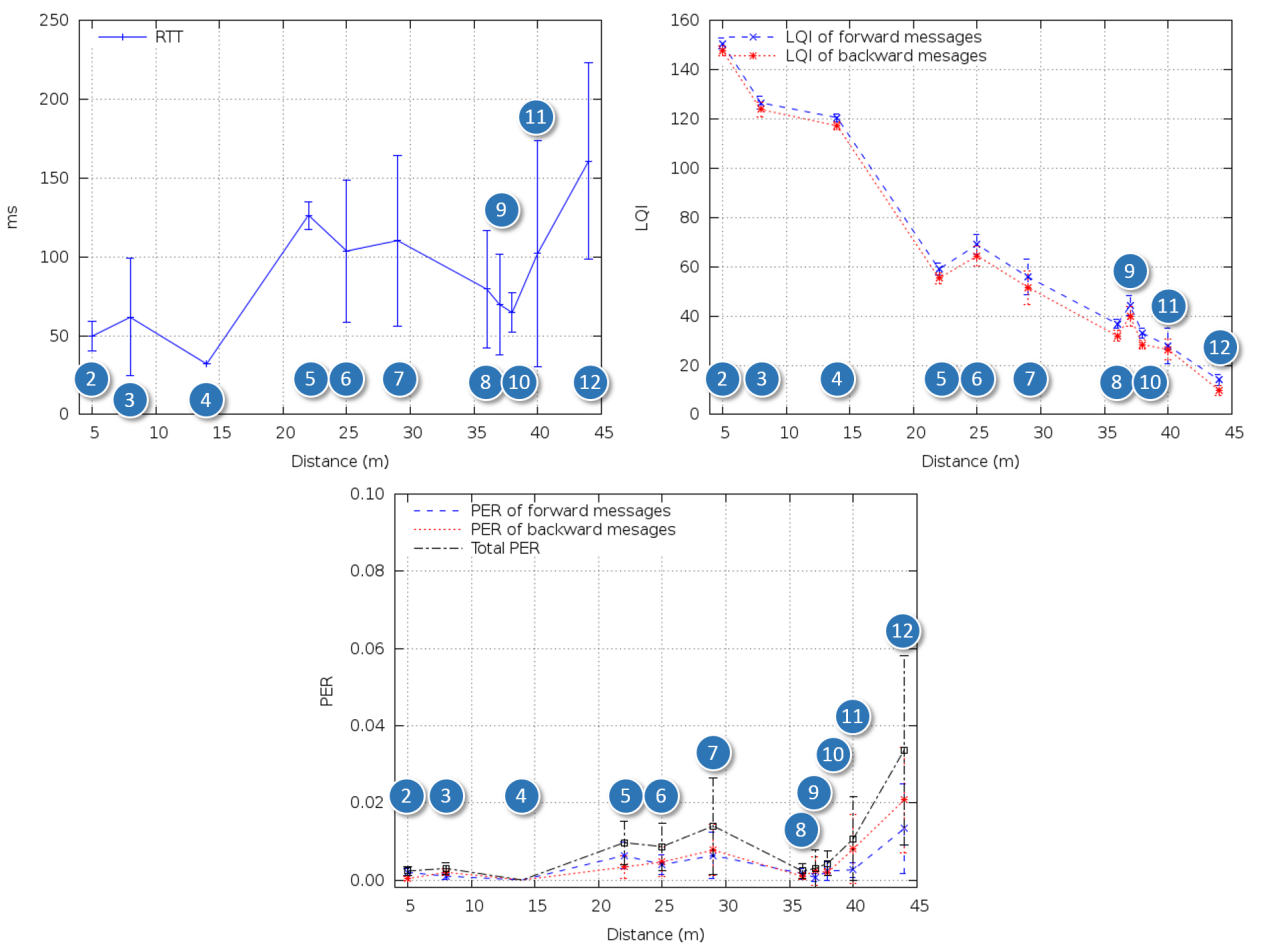
RTT, LQI and PER for in-ship test Scenario 4.

Figure 18 shows the results for Scenario 4. The high variability of the environment is evident.

**Figure 19.**
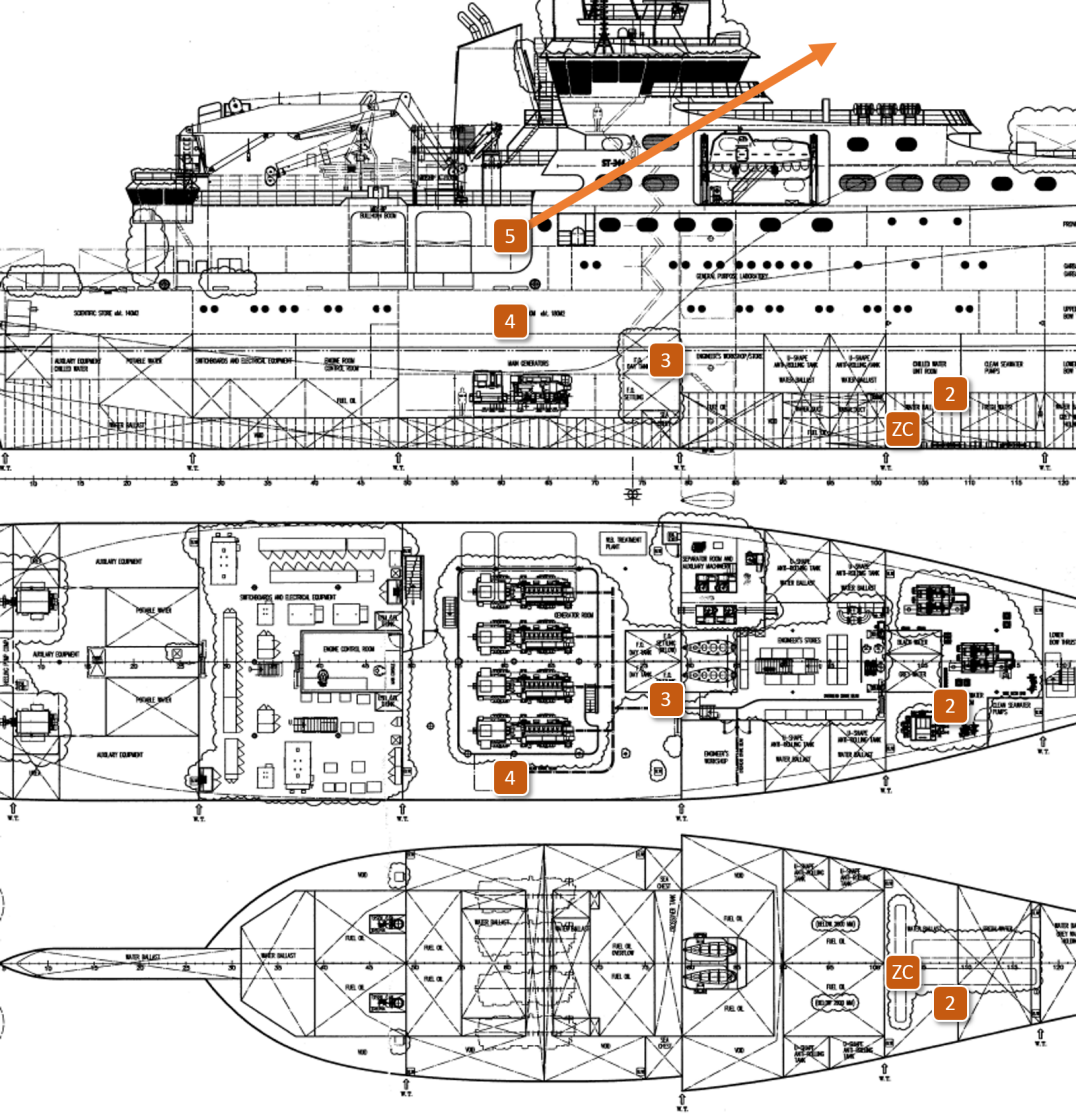
In-ship test Scenario 5: the multihop path from one of the ship holds to a control station outside the vessel.

Finally, in Scenario 5 (Figure 19), a complete multihop path from one of the ship's deepest holds to a control station located 100 m outside the vessel was evaluated. Given the results of the previous tests, we were able to install a few ZRs between the ZC and the ZED with an educated guess, for the messages to reach their destination with sufficient quality. We must remark, again, that unlike for ships in active service, node planning is not possible in this environment.

**Figure 20.**
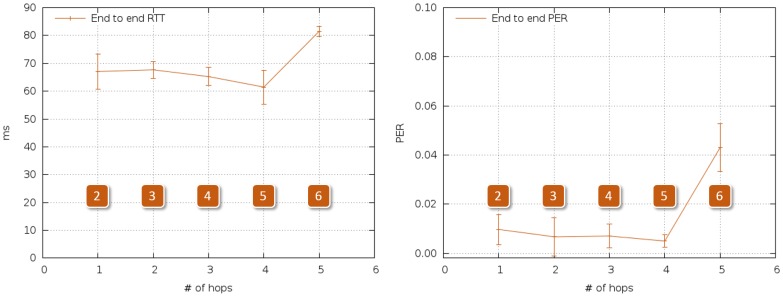
RTT and PER for multihop in-ship tests, test Scenario 5.

As in the case of Figure 20, at most five hops were needed to establish a successful communication between any point inside the ship and the outside. RTT stayed below 90 ms, which we consider negligible for safety protocols, and maximum PER was around 4%, which does not prevent repetitive alerts from reaching the control station in time.

## 5. Conclusions and Future Work

We have presented a system for remote monitoring of toxic gases in shipbuilding to improve worker safety. The system transmits hazard alerts using the IEEE 802.15.4/Zigbee protocol, which was selected for its low power consumption and intrinsic network survivability capabilities.

Using our system, we evaluated the feasibility of transmissions to control stations outside the ship with experimental tests. These tests gave insight into the maximum hop distances and multihop performance of the system, allowing us to transmit data from anywhere inside the ships with relatively simple educated guesses.

Although metal elements greatly hinder wireless transmission, in some of the tests, distances of over 50 m without a line-of-sight were achieved. Workers and machinery present in the vessel introduced some variability in the measurements. Consequently, the confidence intervals of the in-ship results are wider than those of the outdoor reference environment.

The following conclusions, summarized in Table 1, could be drawn from the tests.

- Single-hop tests:
  – Nodes should not be more than 10 m apart in environments with obstacles. This distance could be increased to 30 m if the inner path was clear.
  – RTT and PER increased slightly with distance, as long as retransmission effects were not very pronounced. The complexity of the environment prevented an accurate prediction of their values.
- Multihop tests:
  – In the worst-case scenario, four hops were necessary to extract data frames from the vessel with a low PER (an extra fifth hop was needed to reach the monitoring station outside the ship).
  – RTT increased nonlinearly as new hops were added, but it was always under 100 ms.
  – PER was also higher than in single-hop tests, but it remained under 5%, regardless of the changes in the environment and the disturbances caused by working personnel and machinery.
  – Both RTT and PER values were totally acceptable from the point of view of the end application.

It has been proven experimentally that the proposed system works correctly. ZR placement does not interfere with worker activity, and it is easy to train unskilled personnel to install the system, even though there will logically be variations according to the ship's layout. Future work will seek to minimize the cost and size of the prototypes. Several options will be considered, including existing modular open hardware platforms and *ad hoc* devices based on hardware reference designs.

It would also be interesting to determine available bandwidth in the target scenario and the effect of sending longer data bursts through the network on PER and message delivery delays.

Other extensions of the work presented might include supporting bidirectional short audio messages between the detector and the monitoring station. With this functionality, the worker and the supervisors would be able to communicate directly in the event of an alarm situation.

Finally, it might also be interesting to consider some commonly used auxiliary systems in other environments, such as image sensors and elements that store and transmit images captured after an alarm event.

## Figures and Tables

**Table 1.** Summary of results.

Maximum recommended inter-node distance	10 m
Maximum inter-node distance in the test scenario	63 m
Maximum measured RTT (single-hop, for longest direct transmission path)	193 ms
Maximum measured RTT (multihop, for maximum end-to-end distance)	82 ms
Maximum measured PER (single-hop)	3.6%
Maximum measured PER (multihop)	4.3%
Minimum measured LQI	9.8 dBm
